# Factors associated with the informal use of HIV pre‐exposure prophylaxis in Germany: a cross‐sectional study

**DOI:** 10.1002/jia2.25395

**Published:** 2019-10-03

**Authors:** Uwe Koppe, Ulrich Marcus, Stefan Albrecht, Klaus Jansen, Heiko Jessen, Barbara Gunsenheimer‐Bartmeyer, Viviane Bremer

**Affiliations:** ^1^ Department of Infectious Disease Epidemiology Robert Koch‐Institute Berlin Germany; ^2^ Department of Epidemiology and Health Monitoring Robert Koch‐Institute Berlin Germany; ^3^ Praxis Jessen² + Kollegen Berlin Germany

**Keywords:** PrEP, men who have sex with men, testing, informal PrEP, non‐daily use, affordability

## Abstract

**Introduction:**

Until September 2019, pre‐exposure prophylaxis (PrEP) with tenofovir disoproxil/emtricitabine for HIV prevention was not covered by health insurance plans in Germany, and was only available through private prescriptions with self‐pay or through informal non‐prescription sources. The objective of this study was to investigate the proportion of informal PrEP use among PrEP users and to identify factors of public health relevance that might be associated with informal PrEP use.

**Methods:**

We conducted a cross‐sectional study recruiting PrEP users independent of their PrEP source. Clients from anonymous community testing checkpoints, users of three dating apps for men who have sex with men residing in Germany and users of a PrEP community website, were recruited to complete a short anonymous online survey. Participants were recruited between 24 July and 3 September 2018. The results were analysed using univariable and multivariable logistic regressions.

**Results:**

We recruited 2005 participants currently using PrEP. The median age was 38 years, and 80.3% of the participants identified themselves as male (missing: 19.1%). Overall, 71.6% obtained PrEP through medical services with a private prescription or a clinical trial, and 17.4% obtained PrEP through informal sources (missing: 11.0%). The most common informal sources were ordering online from another country (8.8%), travel abroad (3.6%), and friends (2.5%). Factors associated with informal PrEP use were on demand/intermittent dosing (adjusted OR: 3.5, 95% CI 2.5 to 5.0) and not receiving medical tests during PrEP use (adjusted OR: 3.2, 95% CI 2.0 to 5.2). In addition, informal PrEP users who did not take PrEP daily had a strongly increased risk of starting PrEP without prior medical tests (adjusted stratum‐specific OR = 31.7, 95% CI 4.6 to 219.5).

**Conclusions:**

Informal PrEP use was associated with a higher risk of not getting tested before and during PrEP use, which could lead to HIV infections resistant to tenofovir and emtricitabine if people with undiagnosed HIV use PrEP. Health insurance plans that cover PrEP and the accompanying routine tests could ensure adequate medical supervision of PrEP users and reduce barriers to PrEP use. Our findings strongly support the implementation of PrEP programmes in countries with similar patterns of informal PrEP use.

## 1 Introduction

Pre‐exposure prophylaxis (PrEP), with tenofovir disoproxil/emtricitabine, has been proven to be highly effective in preventing HIV infections in men who have sex with men (MSM) and other risk groups, both in clinical trials and observational studies 1, 2, 3. However, the appropriate use of PrEP requires exclusion of conditions contraindicated for PrEP use, including a pre‐existing HIV infection or compromised kidney function, in addition to regular monitoring of HIV status, kidney function and testing for other sexually transmitted infections (STIs) 4, 5, 6. Current guidelines recommend HIV testing every three months, STI testing every three to six months and kidney function testing every six to twelve months 4, 5. PrEP should only be initiated and maintained in HIV‐negative people to avoid improper antiviral therapy and the emergence of resistance against tenofovir disoproxil/emtricitabine 1, 7. Currently, PrEP is only approved for daily dosing in the European Union 8, but other dosing regimens, such as on‐demand dosing or intermittent dosing, were investigated in clinical trials and are also used in practice in some European countries 9, 10, 11, 12, 13.

In Germany, about 86,100 people currently live with HIV 14. MSM are the most affected group, with approximately 2700 new infections in 2017. PrEP became available in Germany in 2016. However, the costs for the medication and for the recommended tests (e.g. for HIV, STIs, kidney function) were not covered by health insurance plans before September 2019. The initially high out‐of‐pocket costs for obtaining prescription PrEP—more than €800 per month (assuming daily dosing)—brought many users to start obtaining PrEP through informal sources, such as ordering online from sources outside of the European Union 10, 15, 16. It is unclear whether informal PrEP users seek the recommended medical supervision before and during PrEP use, which could increase the risk of improper antiviral therapy in undetected HIV infections and the emergence of HIV‐strains resistant to tenofovir disoproxil/emtricitabine. Between October 2017 and September 2019, generic PrEP was available in Germany for €40–€70 self‐pay per month (assuming daily dosing) with a private prescription from a physician, which increased the number of PrEP users 11, 17, 18. The recommended tests for HIV, STIs and kidney function could be provided by a physician or by anonymous testing sites in some cities but they required self‐pay before September 2019. However, it is unknown how many people were using informal PrEP during this period and how they were medically supervised.

To investigate these questions, we conducted the PrApp study, a cross‐sectional survey recruiting current and former PrEP users in Germany using geolocation dating apps and other community resources. The objective of this analysis was to investigate the proportion of prescription and informal PrEP use in current PrEP users and to identify factors associated with informal PrEP use to inform Public Health strategies.

## 2 Methods

### 2.1 Study design

The PrApp study is a cross‐sectional study that was designed to investigate PrEP use in MSM residing in Germany 19, 20. Clients of anonymous community testing checkpoints, as well as users of three dating apps for MSM (Grindr, Planetromeo and Hornet) and a community website (https://prepjetzt.de/) were recruited using: a) instant messages or banner advertisements, which linked potential participants to an online questionnaire; b) flyers distributed in checkpoints containing a link and a QR code; c) peer‐to‐peer recruitment. App‐advertising was targeted to profiles of users residing in Germany. We aimed to recruit a diverse group of PrEP users irrespective of where they obtained PrEP. Participants were eligible for inclusion if they: a) provided consent for participation and b) were taking PrEP currently or had taken PrEP in the past. Eligible participants completed a short anonymous online survey. The questionnaire was available on mobile phones and desktop computers and was provided using the VOXCO Acuity4Survey platform. The questionnaire was provided in German, English, French, Spanish, Arabic and Turkish. After completion of the survey, participants could choose to enter a lottery drawing for gift certificates. Data were stored and analysed in accordance with German and European data protection laws.

### 2.2 Outcomes and covariates

The main outcome of this study is the proportion of prescription PrEP use and informal PrEP use. Prescription PrEP use was defined as obtaining PrEP medication through medical services in Germany (e.g. German pharmacies (local or online) with a prescription or participation in a clinical trial). Informal PrEP use was defined as any other PrEP source, including ordering online from another country, traveling and buying PrEP abroad, friends, using medication for post‐exposure prophylaxis as PrEP, dealers, sex parties and other sources 21, 22.

The questionnaire used in this study can be found in the appendix (Appendix S1). Self‐reported gender was analysed as “male,” “transgender/non‐binary” (someone whose gender identity does not match the gender assigned at birth 23, 24) and “intersex” (someone whose sex characteristics do not fit the normative criteria of female or male 25). Age was grouped into the following categories: 18 to 29, 30 to 39, 40 to 49 and 50 to 80 years. The country of origin was grouped into a binary variable “Germany” and “outside Germany.” The type of PrEP use was binarized into “daily” and “on demand/intermittent” use, where “on demand use” refers to PrEP use only around sexual activities 13 and “intermittent” refers to daily PrEP use for shorter periods of time interrupted by periods without PrEP use. Time since first PrEP use was grouped into a binary variable of ≤6 months and ≥7 months. Condom use while taking PrEP was binarized into “always/often” and “in about half of the time/sometimes/never.” The number of anal sex partners within the last six months was grouped as zero to three partners, four to ten partners and >10 partners. The stratification for the number of pills taken per month was based on strata published by Molina *et al*. 13 and simplified into three categories.

Answers to reasons for taking PrEP were grouped as follows (multiple answers per person were allowed): (1) “Protection when condoms are not used” includes anyone with an affirmative answer to “I don't want to use condoms and still want to protect myself”, “My partner doesn't want to use condoms and I still want to protect myself”, “Sex without a condom is expected by my peers and I still want to protect myself”, “Sometimes condoms aren't available and I still want to be protected”, “It's more convenient since I don't have to talk about or negotiate condom use” or free text answers with similar statements; (2) “Protection in addition to condoms” includes anyone with a positive answer to “I want to protect myself against HIV in case the condom breaks” or “I use condoms and I want additional protection”; (3) “Problems using condoms during sex” includes any positive answer to “I cannot get an erection when I use a condom but I still want to protect myself” or free text answers with similar statements; (4) “Serodiscordant partnership” includes anyone with a positive answer to “My partner is HIV positive and I want to protect myself”.

### 2.3 Statistical analysis

The answers to categorical items in the questionnaire are displayed as absolute numbers or proportions. Continuous variables are shown using medians and interquartile ranges.

Factors associated with informal PrEP use were investigated using univariable and multivariable logistic regressions. Co‐variates included age, annual gross income, country of origin, type of PrEP use, time since first PrEP use, average number of pills taken per month, affordability of generic PrEP, tests before starting PrEP, tests while taking PrEP, number of anal sex partners within the last six months, condom use while taking PrEP and communication of PrEP use on online profile. Participants with missing data for any variable included in the final multivariable regression model were excluded from both uni‐ and multivariable analyses (full case analysis). Interactions were analysed using likelihood ratio tests and calculating stratum‐specific effect estimates.

As a sensitivity analysis, we performed the analyses excluding patients that received PrEP through a clinical trial since their PrEP use and testing behaviour might differ from other PrEP users. In addition, participants receiving prescription PrEP through a German online pharmacy might have misclassified themselves to the informal PrEP use category “Internet/ordered online from another country”. We performed a sensitivity analysis excluding participants from this category to investigate the robustness of our findings.

### 2.4 Ethical approval

The study protocol was approved by the ethics commission of the Berlin Chamber of Physicians (Ref: Eth‐14/18).

## 3 Results

Between 24 July and 3 September 2018, we recruited 2005 participants who were using PrEP (Appendix S2). The median age was 38 years and 80.3% identified themselves as male (Table 1).

**Table 1:** Baseline data of participants

	Participants
Total (n)	2005
Age (years)	
Median (IQR)	38 (31 to 45)
18 to 29, n (%)	330 (16.5)
30 to 39, n (%)	606 (30.2)
40 to 49, n (%)	475 (23.7)
50 to 80, n (%)	213 (10.6)
Missing, n (%)	381 (19.0)
Gender, n (%)	
Male	1609 (80.3)
Transgender/non‐binary	10 (0.5)
Intersex	4 (0.2)
Missing	382 (19.1)
Recruited through, n (%) (multiple answers allowed)	
Dating apps	1293 (64.5)
Community website	142 (7.1)
Anonymous checkpoint	40 (2.0)
Friends	619 (30.9)
Missing	428 (21.3)
Reasons for taking PrEP, n (%) (multiple answers allowed)	
Protection when condoms are not used	1567 (78.2)
Protection in addition to condoms	791 (39.5)
Problems using condoms during sex	554 (27.6)
HIV serodiscordant partnership	158 (7.9)
Other reason/missing	12 (0.6)
Sources of PrEP	
Medical services with private prescription or clinical trial	1436 (71.6)
Online order from another country	177 (8.8)
Travel to foreign country	73 (3.6)
Friends	51 (2.5)
Using medication from post‐exposure prophylaxis	17 (0.9)
Dealers	15 (0.7)
Sex parties	15 (0.7)
Other	1 (0.1)
Missing	220 (11.0)

IQR, interquartile range; PrEP, pre‐exposure prophylaxis.

Most participants were recruited through dating apps (64.5%), followed by friends (30.9%), a community website (7.1%) and checkpoints (2.0%). The most common reason for taking PrEP was to have protection when condoms are not used, followed by wanting protection in addition to condoms, having problems using condoms and being in a serodiscordant partnership.

While obtaining PrEP through medical services was most common, 17.4% obtained PrEP through informal sources (Table 1). Informal PrEP users were more likely than prescription PrEP users to have first used PrEP longer than six months ago (adjusted OR (aOR) = 2.6, 95% CI 1.8 to 3.7) (Table 2). In addition, informal PrEP users were more likely to use PrEP on‐demand or intermittently compared to prescription PrEP users (aOR = 3.5, 95% CI 2.5 to 5.0).

**Table 2:** Uni‐ and multivariable analysis to investigate factors associated with informal PrEP use

	Prescription PrEP n (%)	Informal PrEP n (%)	Univariable analysis^a^		Multivariable analysis^b^	
			OR (95% CI)	*p*‐value	OR (95% CI)	*p*‐value
Total	1436 (100)	348 (100)				
Time since first PrEP use						
≤6 months	686 (47.8)	91 (26.2)	1		1	
≥7 months	656 (45.7)	218 (62.6)	2.5 (1.8 to 3.4)	< 0.001	2.6 (1.8 to 3.7)	< 0.001
Missing	94 (6.6)	39 (11.2)	‐			
Type of PrEP use						
Daily	1034 (72.0)	139 (40.0)	1		1	
On demand/intermittent	311 (21.7)	168 (48.3)	4.3 (3.2 to 5.8)	< 0.001	3.5 (2.5 to 5.0)	< 0.001
Missing	91 (6.3)	41 (11.8)	‐			
Tests before starting PrEP (e.g. HIV, STI, kidney function)						
Yes	1391 (96.9)	289 (83.1)	1		1	
No	27 (1.9)	43 (12.4)	7.3 (3.9 to 13.9)	< 0.001	3.0 (1.4 to 6.6)	0.006
Missing	18 (1.3)	16 (4.6)	‐			
Tests while taking PrEP (e.g. HIV, STI, kidney function)						
Yes	1274 (88.7)	223 (64.1)	1		1	
No	96 (6.7)	96 (27.6)	6.1 (4.2 to 8.9)	< 0.001	3.2 (2.0 to 5.2)	< 0.001
Missing	66 (4.6)	29 (8.3)	‐			
Country of origin (%)						
Germany	913 (63.6)	164 (47.1)	1		1	
Outside Germany	269 (18.7)	99 (28.5)	2.0 (1.5 to 2.8)	< 0.001	2.3 (1.6 to 3.2)	< 0.001
Missing	254 (17.7)	85 (24.4)	‐			
Number of anal sex partners within the last 6 months, n (%)						
0 to 3	190 (13.2)	49 (14.1)	1.2 (0.7 to 1.8)	0.509	1.0 (0.6 to 1.7)	0.978
4 to 10	417 (29.0)	90 (25.9)	1		1	
>10	711 (49.5)	164 (47.1)	1.1 (0.8 to 1.5)	0.584	1.2 (0.8 to 1.7)	0.466
Missing	118 (8.2)	45 (12.9)	‐			
Condom use while taking PrEP						
Always/often	283 (19.7)	55 (15.8)	1		1	
In about half of the times/sometimes/never	1046 (72.8)	248 (71.3)	1.3 (0.9 to 1.9)	0.203	1.4 (0.9 to 2.1)	0.199
Missing	107 (7.5)	45 (12.9)	‐			
Is PrEP for a price of €50 to €70 per month affordable?						
Yes	950 (66.2)	183 (52.6)	1		1	
Yes, but it is difficult to manage	417 (29.0)	91 (26.2)	1.4 (1.0 to 1.9)	0.057	1.6 (1.0 to 2.4)	0.040
No	49 (3.4)	57 (16.4)	7.2 (4.4 to 12.0)	< 0.001	7.1 (3.8 to 13.1)	< 0.001
Missing	20 (1.4)	17 (4.9)	‐			
Communication of PrEP on online profile						
Yes	655 (45.6)	131 (37.6)	1		1	
No, but mentions it while chatting	453 (31.6)	122 (35.1)	1.3 (1.0 to 1.8)	0.082	1.0 (0.7 to 1.4)	0.993
No	217 (15.1)	53 (15.2)	1.2 (0.8 to 1.8)	0.423	0.9 (0.5 to 1.4)	0.588
Missing	111 (7.7)	42 (12.1)	‐			
Age						
18 to 29 years	253 (17.6)	77 (22.1)	1.4 (1.0 to 2.1)	0.077	1.0 (0.6 to 1.6)	0.959
30 to 39 years	514 (35.8)	92 (26.4)	1		1	
40 to 49 years	377 (26.3)	98 (28.2)	1.2 (0.8 to 1.7)	0.295	1.3 (0.9 to 1.9)	0.206
50 to 80 years	177 (12.3)	35 (10.1)	0.9 (0.5 to 1.4)	0.597	0.8 (0.4 to 1.4)	0.410
Missing	115 (8.0)	46 (13.2)				
Annual gross income, n (%)						
<€30,000	320 (22.3)	79 (22.7)	1		1	
€30,000 to €39,000	217 (15.1)	55 (15.8)	0.8 (0.5 to 1.2)	0.245	1.6 (0.9 to 2.7)	0.095
€40,000 to €49,000	191 (13.3)	35 (10.1)	0.7 (0.4 to 1.1)	0.156	1.3 (0.7 to 2.3)	0.407
€50,000 to €59,000	144 (10.0)	27 (7.8)	0.7 (0.4 to 1.1)	0.142	1.6 (0.8 to 3.2)	0.153
€60,000 to €69,000	109 (7.6)	31 (8.9)	1.2 (0.7 to 2.0)	0.497	2.4 (1.3 to 4.6)	0.008
≥€70,000	265 (18.5)	55 (15.8)	0.8 (0.6 to 1.3)	0.386	1.7 (1.0 to 3.1)	0.072
Missing	190 (13.2)	66 (19.0)	‐			

CI, confidence interval; HIV, human immunodeficiency virus; OR, odds ratio, PrEP, PRE‐exposure prophylaxis; STI, sexually transmitted infection.

^a^Univariable logistic regression model including 1089 participants with formal and 230 participants with informal PrEP use, *p*‐values derived from Wald test; ^b^multivariable logistic regression model including 1089 participants with formal and 230 participants with informal PrEP use adjusting for age, annual gross income, time since first PrEP use, type of PrEP use, tests before starting PrEP, tests while taking PrEP, country of origin, number of anal sex partners within the last six months, condom use while taking PrEP, affordability of PrEP, communication of PrEP use online; *p*‐values derived from Wald test.

Informal PrEP users were more likely to use PrEP without undergoing the recommended medical tests before starting PrEP (aOR = 3.0, 95% CI 1.4 to 6.6) or during PrEP use (aOR = 3.2, 95% CI 2.0 to 5.2). Further stratification revealed that not getting tested before starting PrEP was strongly associated with informal PrEP users taking on demand or intermittent PrEP (adjusted stratum‐specific OR = 31.7, 95% CI 4.6 to 219.5) in contrast to informal PrEP users taking daily PrEP (Table 3).

**Table 3:** Stratum‐specific odds ratios of multivariable logistic regression analysis investigating factors associated with informal PrEP use

	Daily PrEP use		On demand/intermittent PrEP use		Likelihood ratio test for interaction
	OR (95% CI)	*p*‐value	OR (95% CI)	*p*‐value	*p*‐value
Tests before starting PrEP (e.g. HIV, STI, kidney function)					
Yes	1		1		0.013
No	0.6 (0.1 to 3.3)	0.567	31.7 (4.6 to 219.5)	<0.001	
Tests while taking PrEP (e.g. HIV, STI, kidney function)					
Yes	1		1		0.833
No	3.5 (1.6 to 7.8)	0.002	3.2 (1.3 to 7.8)	0.009	
Is PrEP for a price of €50 to €70 per month affordable?					
Yes	1		1		0.058
Yes, but it is difficult to manage	2.0 (1.2 to 3.4)	0.008	2.5 (1.4 to 4.6)	0.003	
No	11.5 (5.3 to 25.3)	<0.001	1.6 (0.6 to 4.4)	0.407	

Multivariable logistic regression model adjusting for age, annual gross income, country of origin, type of PrEP use, time since first PrEP use, affordability of generic PrEP, tests before starting PrEP, tests during PREP use, number of anal sex partners within the last six months, condom use, communication of PrEP use online; *p*‐values: Wald test. CI, confidence interval; HIV, human immunodeficiency virus; OR, odds ratio; PrEP, pre‐exposure prophylaxis; STI, sexually transmitted infection.

Not getting tested during PrEP use was associated with informal PrEP use regardless of participants using daily or on demand/intermittent PrEP (Table 3). More informal PrEP users stated that their country of origin was not Germany and that generic PrEP for €50–€70 per month was not affordable for them (Table 2). We found weak evidence that the answers regarding the affordability of PrEP differed across strata of daily versus on demand/intermittent PrEP use (Table 3). In daily PrEP users, we found that informal PrEP users were much more likely than prescription PrEP users to state that they perceived generic PrEP to be unaffordable (adjusted stratum‐specific OR = 11.5, 95% CI 5.3 to 25.3). In non‐daily PrEP users, we did not find evidence for the association between informal PrEP use and perceiving generic PrEP to be unaffordable.

Condom use was low, as 72.5% stated that they used condoms on half or less of the occasions they had anal intercourse (Table 2). We did not find a difference in condom use between prescription and informal PrEP users. Since taking PrEP, 50.1% of the participants indicated using condoms less often than before, and 21.3% stopped using condoms completely (Appendix S3).

PrEP use was communicated on the online dating profile of 44.6% of the participants, and an additional 32.2% indicated mentioning PrEP use in chats (Table 2). The proportion of participants taking PrEP less than 26 days per month was higher in informal PrEP users (Appendix S3). However, after adjusting for confounding variables, we did not find an association of this variable with informal PrEP use (adjusted OR = 1.4, 95% CI 0.8 to 2.6); thus, the variable was excluded from the multivariable regression model.

In the final multivariable model, we did not find differences between prescription PrEP users and informal PrEP users regarding age, annual gross income, number of anal sex partners within the last six months, condom use or communicating PrEP use online (Table 2). Sensitivity analyses excluding participants receiving PrEP through a clinical trial (Appendix S4) or excluding participants receiving informal PrEP online (Appendix S5) yielded similar results.

## 4 Discussion

In this study, which investigated PrEP use in a sample of German PrEP users recruited between 24 July and 3 September 2018 (i.e. nine to ten months after sharp price reductions for generic PrEP medication in Germany), we show that 71.6% of the participants used prescription PrEP and 17.4% used informal sources (missing: 11.0%). Informal PrEP users were more likely to use PrEP on‐demand or intermittently and more likely to forgo medical testing during PrEP use. In addition, informal PrEP users using on‐demand or intermittent PrEP had a high risk of not getting medical tests before initiating PrEP. Individuals that avoid getting the recommended tests before and during PrEP increase the risk of using PrEP in case of an undiagnosed HIV infection, which consequently increases the risk of developing tenofovir disoproxil/emtricitabine‐resistant HIV infections.

We were able to recruit a large sample of PrEP users to comprehensively investigate the extent of informal PrEP use and associated factors. The recruited population is similar to previously published studies regarding age, a high number of sexual partners and low condom use 9, 10, 11, 12, 16, 18, 26, 27. Surveys from France and Australia reported informal PrEP use predominantly in younger populations 16, 27, which might be due to recruitment differences. The proportion of prescription PrEP use was higher in our study (71.6%) compared to a German online survey from 2016 (29.2%) and a study in Berlin from October 2017–April 2018 (44.4%) 11, 17. Another study found an even higher proportion of prescription PrEP users in Germany (98%), which might be due to differences in recruitment and the underlying study population 18. This increase in prescription PrEP use might reflect access to the more affordable generic PrEP option, which became available in October 2017. These observations are in line with our findings that more informal PrEP users had been using PrEP for more than 12 months, which likely started before generic PrEP became available in Germany.

Regarding the type of PrEP use, previous studies in Belgium, the Netherlands and Australia found that between 23.5% and 48% of the participants preferred non‐daily PrEP 12, 28, 29, 30, 31. A single‐centre study in France found the preference for on‐demand PrEP to be as high as 75.6% 9. In our study, we found the proportion of on‐demand/intermittent PrEP users to be on the lower end of this spectrum (23.9%). It will be interesting to monitor the development of daily and on‐demand PrEP use in future surveys.

Regarding the reasons for PrEP use, a qualitative study identified different types of informal PrEP users: people who want to avoid condom usage and others who use PrEP as additional protection in case a condom breaks 32. Our study corroborated these findings, showing that the majority the participants used PrEP for protection in case condoms were not used, but about 40% also indicated that they preferred protection in addition to condoms.

The appropriate use of PrEP requires medical testing before and during use to avoid PrEP use in people with undiagnosed HIV infections and to ensure the timely diagnosis of other STIs 4, 5. In Germany, recommended routine testing during PrEP use is not covered by health insurance plans, which might prevent some PrEP users from getting the recommended tests. In addition, people using informal PrEP might lack financial resources or even the knowledge where to obtain appropriate medical supervision, and anonymous testing sites might not offer all the tests. In our study, the majority of people stated that they obtained medical tests before and during PrEP use. The testing frequency in prescription‐PrEP users in our study is higher than results found in two previously published studies using data collected between 2011 and 2017 33, 34, which might be due to differences in data capture and more recent dissemination of testing guidelines. However, we did identify that informal PrEP users were more likely to forgo testing before or during PrEP use. Recurrent out‐of‐pocket costs for these tests, a lack of information that these tests are needed and/or a lack of knowledge where to get access to these tests are among the possible reasons informal PrEP users do not get tested. We did not collect data on the causal factors between informal use and insufficient testing behaviour. This should be investigated in the future.

Informal PrEP users were more likely to state that they perceived generic PrEP for €50–€70 per month (assuming daily dosing) to be unaffordable. Since the price of PrEP through online sources is still lower than that of generic PrEP, people with less disposable income might stay on informal PrEP. Stratification by the type of PrEP use showed that finding generic PrEP unaffordable was strongly associated with informal PrEP use in daily users. Interestingly, we did not find this association for on‐demand/intermittent users, indicating that daily PrEP use without health insurance coverage is likely too expensive for some, and the high cost prevents access to all people who could benefit from it. This is noticeable in light of recent results indicating that in some populations daily PrEP might allow for more intercourse events to be protected against HIV 35, 36. In contrast, about half of the informal PrEP users indicated that generic PrEP would be affordable to them. In our study, we did not investigate reasons why they still opted to obtain informal PrEP. Possible reasons for the continued use of informal PrEP could include the inconvenience of managing doctors’ appointments and filling prescriptions, difficulties with finding a physician willing to prescribe PrEP and/or the stigma associated with PrEP.

Since September 2019, PrEP, as well as the accompanying tests, are covered by statutory health insurances covering about 90% of the German population, with the remainder being covered by private health insurances or self‐pay. In light of our results, we strongly support this initiative to ensure that all people with HIV prevention needs can access PrEP under medical supervision, regardless of their financial background and to encourage other countries with similar patterns of informal use to consider similar measures.

The strength of this study is that we were able to recruit a large sample of current PrEP users, as small sample sizes were a common limitation of previous studies 11, 17. In addition, we were able to recruit participants independent of their PrEP source using an easily accessible, anonymous online survey. Since informal PrEP use implies obtaining PrEP medication from “unauthorised” sources, participants may not have been as open to report on their experiences in a face‐to‐face setting.

Some limitations of this study need to be considered. Since most of the participants were recruited online, it is unclear how generalizable the results are for PrEP users not using online platforms. Also, since we did not have any information on people who were not willing to participate in the study, we were not able to investigate potential selection bias. Moreover, some people who informally use antiretroviral medication to prevent HIV infections might not refer to the medication as PrEP, and therefore did not participate in this survey. Thus, we do not know if the results of this study apply to this group.

Additionally, since the study results relied on self‐reported information by the participants, they might be subject to several information biases. People might be likely to provide more socially acceptable answers on sensitive topics, such as sexual behaviour and condom use. However, our data show that we were able to recruit people that indicated risky sexual behaviour; so, we did not expect this to have a major effect on our results. In addition, the provided answers might be subject to recall bias. Since we did not expect either effect to be dependent on the source of PrEP, this effect would be non‐differential between the groups and bias our effect estimates towards the null value. Participants might not want to disclose their true source of PrEP since it might be illegal. In this case, some people with informal sources would be misclassified as using prescription PrEP. This would make the groups more alike and bias our effect estimates towards the null value. Considering these factors, our effect estimates for factors associated with informal PrEP use might be underestimates. In addition, participants receiving prescription PrEP from online pharmacies might have misclassified themselves into the informal PrEP category “online order from another country.” This would also make the categories more alike and bias our effect estimates towards the null value. However, a sensitivity analysis excluding participants ordering PrEP online yielded similar results; thus, we do not believe our findings were substantially impacted by this factor.

## 5 Conclusions

Our study shows that informal PrEP use is associated with a higher risk of not getting tested before and during PrEP use, which can lead to HIV infections resistant to tenofovir disoproxil/emtricitabine if PrEP is used in people with undiagnosed HIV. A substantial proportion of PrEP users, especially informal PrEP users, struggle with the out‐of‐pocket costs for PrEP, potentially impairing access to this prevention tool. Our data strongly support that PrEP and the corresponding medical tests should be covered by health insurance plans to ensure proper use regardless of the financial background of PrEP users.

## Competing interests

HJ received payment for study cost from Gilead Sciences Inc. The other authors declare no conflicts of interest. This study was promoted by the dating app providers (Grindr, Planetromeo, Hornet), the prepjetzt.de website and the community checkpoints free of charge.

## Authors’ contributions

UK, UM, KJ, HJ, BGB and VB designed the study. UK and SA were involved in data curation. UK coordinated the study, performed the analysis and wrote the first draft. All authors reviewed and contributed to the manuscript.

## Supporting information


**Appendix S1.** Questionnaire.
**Appendix S2.** Recruitment of participants.
**Appendix S3.** Supplemental data on participants with formal and informal PrEP use.
**Appendix S4.** Investigating factors associated with informal PrEP use excluding participants receiving PrEP through clinical trials.
**Appendix S5.** Investigating factors associated with informal PrEP use excluding participants ordering informal PrEP through online sources.

## References

[1] Riddell Jt , Amico KR , Mayer KH . HIV preexposure prophylaxis: a review. JAMA. 2018;319(12):1261–8. 29584848 10.1001/jama.2018.1917

[2] Desai M , Field N , Grant R , McCormack S . Recent advances in pre‐exposure prophylaxis for HIV. BMJ. 2017;359:j5011. 29229609 10.1136/bmj.j5011 PMC6020995

[3] Grulich AE , Guy R , Amin J , Jin F , Selvey C , Holden J , et al. Population‐level effectiveness of rapid, targeted, high‐coverage roll‐out of HIV pre‐exposure prophylaxis in men who have sex with men: the EPIC‐NSW prospective cohort study. Lancet HIV. 2018;5(11):e629–37. 30343026 10.1016/S2352-3018(18)30215-7

[4] Centers for Disease Control and Prevention . US Public Health Service: preexposure prophylaxis for the prevention of HIV infection in the United States—2017 Update: a clinical practice guideline. 2018 [cited 2018 Oct 19]. Available from: https://wwwcdcgov/hiv/pdf/risk/prep/cdc-hiv-prep-guidelines-2017pdf

[5] Deutsche AIDS Gesellschaft e.V. (DAIG) . Deutsch‐Östereichische Leitlinien zur HIV‐Präexpositionsprophylaxe. AWMF‐Register‐Nr: 055‐008. 2018.

[6] Hoornenborg E , Krakower DS , Prins M , Mayer KH . Pre‐exposure prophylaxis for MSM and transgender persons in early adopting countries. AIDS. 2017;31(16):2179–91. 28991023 10.1097/QAD.0000000000001627 PMC5812254

[7] Fonner VA , Dalglish SL , Kennedy CE , Baggaley R , O'Reilly KR , Koechlin FM , et al. Effectiveness and safety of oral HIV preexposure prophylaxis for all populations. AIDS. 2016;30(12):1973–83. 27149090 10.1097/QAD.0000000000001145 PMC4949005

[8] European Medicines Agency . Truvada ‐ Summary of product characteristics. [cited 2019 May 17]. Available from: https://www.ema.europa.eu/en/documents/product-information/truvada-epar-product-information_en.pdf

[9] Noret M , Balavoine S , Pintado C , Siguier M , Brun A , Bauer R , et al. Daily or on‐demand oral tenofovir disoproxil fumarate/emtricitabine for HIV pre‐exposure prophylaxis: experience from a hospital‐based clinic in France. AIDS. 2018;32(15):2161–9. 30212403 10.1097/QAD.0000000000001939

[10] Wang X , Nwokolo N , Korologou‐Linden R , Hill A , Whitlock G , Day‐Weber I , et al. InterPrEP: internet‐based pre‐exposure prophylaxis with generic tenofovir disoproxil fumarate/emtrictabine in London ‐ analysis of pharmacokinetics, safety and outcomes. HIV Med. 2018;19(1):1–6. 28657199 10.1111/hiv.12528

[11] Werner RN , Gaskins M , Ahrens J , Jessen H , Kutscha F , Mosdzen R , et al. Knowledge and use of HIV pre‐exposure prophylaxis among men who have sex with men in Berlin ‐ A multicentre, cross‐sectional survey. PLoS ONE. 2018;13:e0204067. 30212547 10.1371/journal.pone.0204067 PMC6136827

[12] Reyniers T , Nostlinger C , Laga M , De Baetselier I , Crucitti T , Wouters K , et al. Choosing between daily and event‐driven pre‐exposure prophylaxis: results of a Belgian PrEP demonstration project. J Acquir Immune Defic Syndr. 2018;79(2):186–94. 29975211 10.1097/QAI.0000000000001791

[13] Molina JM , Capitant C , Spire B , Pialoux G , Cotte L , Charreau I , et al. On‐Demand Preexposure Prophylaxis in Men at High Risk for HIV‐1 Infection. N Engl J Med. 2015;373(23):2237–46. 26624850 10.1056/NEJMoa1506273

[14] Robert Koch‐Institut . Estimation of the number of new HIV infections and the total number of people living with HIV in Germany. Epid Bull. 2018;47:509–22.

[15] Paparini S , Nutland W , Rhodes T , Nguyen VK , Anderson J . DIY HIV prevention: formative qualitative research with men who have sex with men who source PrEP outside of clinical trials. PLoS ONE. 2018;13:e0202830. 30138482 10.1371/journal.pone.0202830 PMC6107262

[16] Zablotska IB , Prestage G , de Wit J , Grulich AE , Mao L , Holt M . The informal use of antiretrovirals for preexposure prophylaxis of HIV infection among gay men in Australia. J Acquir Immune Defic Syndr. 2013;62(3):334–8. 23187947 10.1097/QAI.0b013e31827e854a

[17] Spinner CD , Hanhoff N , Krznaric I , Knecht G , Kuemmerle T , Ruesenberg R , et al. 2016 PREP attitudes in Germany: high awareness and acceptance in MSM at risk of HIV. Infection. 2018;46(3):405–8. 29478091 10.1007/s15010-018-1127-3

[18] Mysior P , Perea I , Brillen A‐L , Bartmeyer B , Bremer V , Tenberken U , et al. Pre‐exposure prophylaxis (PreP) implementation, uptake and retention in Germany ‐ results of the PRIDE study. 22nd International AIDS Conference Amsterdam. 2018:wePec240.

[19] Koppe U , Marcus U , Albrecht S , Jansen K , Jessen H , Gunsenheimer‐Bartmeyer B , et al. Risk factors associated with non‐prescription use of HIV pre‐exposure prophylaxis. Conference on Retroviruses and Opportunistic Infections (CROI). 2019.

[20] Koppe U , Marcus U , Albrecht S , Jansen K , Jessen H , Gunsenheimer‐Bartmeyer B , et al. Stopping HIV pre‐exposure prophylaxis ‐ reasons and implications. Conference on Retroviruses and Opportunistic Infections (CROI) 2019.

[21] Brisson J . Ethical public health issues for the use of informal PrEP. Glob Public Health. 2018;13(10):1382–7. 28868979 10.1080/17441692.2017.1373139

[22] Brisson J , Ravitsky V , Williams‐Jones B . Informal PrEP: an emerging need for nomenclature. Lancet Public Health. 2019;4(2):e83. 30578132 10.1016/S2468-2667(18)30254-8

[23] Bauer GR , Braimoh J , Scheim AI , Dharma C . Transgender‐inclusive measures of sex/gender for population surveys: mixed‐methods evaluation and recommendations. PLoS ONE. 2017;12:e0178043. 28542498 10.1371/journal.pone.0178043 PMC5444783

[24] Richards C , Bouman WP , Seal L , Barker MJ , Nieder TO , T'Sjoen G . Non‐binary or genderqueer genders. Int Rev Psychiatry. 2016;28(1):95–102. 26753630 10.3109/09540261.2015.1106446

[25] Cresti M , Nave E , Lala R . Intersexual births: the epistemology of sex and ethics of sex assignment. J Bioeth Inq. 2018;15(4):557–68. 30367362 10.1007/s11673-018-9880-7

[26] Holt M , Lea T , Mao L , Kolstee J , Zablotska I , Duck T , et al. Community‐level changes in condom use and uptake of HIV pre‐exposure prophylaxis by gay and bisexual men in Melbourne and Sydney, Australia: results of repeated behavioural surveillance in 2013‐17. Lancet HIV. 2018;5:e448–56. 29885813 10.1016/S2352-3018(18)30072-9

[27] Sagaon‐Teyssier L , Suzan‐Monti M , Demoulin B , Capitant C , Lorente N , Preau M , et al. Uptake of PrEP and condom and sexual risk behavior among MSM during the ANRS IPERGAY trial. AIDS Care. 2016;28 Suppl 1:48–55. 26883400 10.1080/09540121.2016.1146653 PMC4828609

[28] Cornelisse VJ , Lal L , Price B , Ryan KE , Bell C , Owen L , et al. Interest in switching to on‐demand HIV Pre‐Exposure Prophylaxis (PrEP) among Australian users of daily PrEP: an online survey. Open Forum Infect Dis. 2019;6(7):ofz287. 31304192 10.1093/ofid/ofz287 PMC6612821

[29] Coyer L , van Bilsen W , Bil J , Davidovich U , Hoornenborg E , Prins M , et al. Pre‐exposure prophylaxis among men who have sex with men in the Amsterdam Cohort Studies: Use, eligibility, and intention to use. PLoS ONE. 2018;13:e0205663. 30312336 10.1371/journal.pone.0205663 PMC6185853

[30] Hoornenborg E , Achterbergh RC , van der Loeff MFS , Davidovich U , van der Helm JJ , Hogewoning A , et al. Men who have sex with men more often chose daily than event‐driven use of pre‐exposure prophylaxis: baseline analysis of a demonstration study in Amsterdam. J Int AIDS Soc. 2018;21:e25105. 29603900 10.1002/jia2.25105 PMC5878413

[31] Vaccher SJ , Gianacas C , Templeton DJ , Poynten IM , Haire BG , Ooi C , et al. Baseline Preferences for Daily, Event‐Driven, or Periodic HIV Pre‐Exposure Prophylaxis among Gay and Bisexual Men in the PRELUDE Demonstration Project. Front Public Health. 2017;5:341. 29326917 10.3389/fpubh.2017.00341 PMC5736869

[32] Rivierez I , Quatremere G , Spire B , Ghosn J , Rojas Castro D . Lessons learned from the experiences of informal PrEP users in France: results from the ANRS‐PrEPage study. AIDS Care. 2018;30(sup2):48–53. 29848005 10.1080/09540121.2018.1468014

[33] Huang YA , Tao G , Samandari T , Hoover KW . Laboratory Testing of a Cohort of Commercially Insured Users of HIV Preexposure Prophylaxis in the United States, 2011‐2015. J Infect Dis. 2018;217(4):617–21. 29145597 10.1093/infdis/jix595

[34] Spinelli MA , Scott HM , Vittinghoff E , Liu AY , Morehead‐Gee A , Gonzalez R , et al. Provider adherence to pre‐exposure prophylaxis monitoring guidelines in a large primary care network. Open Forum Infect Dis. 2018;5(6):ofy099. 29977959 10.1093/ofid/ofy099 PMC6016415

[35] Bekker LG , Roux S , Sebastien E , Yola N , Amico KR , Hughes JP , et al. Daily and non‐daily pre‐exposure prophylaxis in African women (HPTN 067/ADAPT Cape Town Trial): a randomised, open‐label, phase 2 trial. Lancet HIV. 2018;5(2):e68–78. 28986029 10.1016/S2352-3018(17)30156-X PMC6107917

[36] Grant RM , Mannheimer S , Hughes JP , Hirsch‐Moverman Y , Loquere A , Chitwarakorn A , et al. Daily and nondaily oral preexposure prophylaxis in men and transgender women who have sex with men: the human immunodeficiency virus prevention trials network 067/ADAPT study. Clin Infect Dis. 2018;66(11):1712–21. 29420695 10.1093/cid/cix1086 PMC5961078

