# Supplementary material for: Factors associated with the informal use of HIV pre‐exposure prophylaxis in Germany: a cross‐sectional study
Source: J Int AIDS Soc. 2019 Oct 3;22(10):e25395. doi: 10.1002/jia2.25395 (PMC6776824; doi:10.1002/jia2.25395)
Supplement: Supplementary file 1 — Appendix S1. Questionnaire. Appendix S2. Recruitment of participants. Appendix S3. Supplemental data on participants with formal and informal PrEP use. Appendix S4. Investigating factors associated with informal PrEP use excluding participants receiving PrEP through clinical trials. Appendix S5. Investigating factors associated with informal PrEP use excluding participants ordering informal PrEP through online sources. [file JIA2-22-e25395-s001.docx]

# Appendix: Risk factors associated with non-prescription use of HIV pre-exposure prophylaxis

# Overview

Appendix S1 – Questionnaire

Appendix S2 – Recruitment of participants

Appendix S3 - Supplemental data on participants with formal and informal PrEP use

Appendix S4 - Investigating factors associated with informal PrEP use excluding participants receiving PrEP through clinical trials

Appendix S5 - Investigating factors associated with informal PrEP use excluding participants ordering informal PrEP through online sources

## Appendix S1: Questionnaire

| **Question** | **Answer options** |
| --- | --- |
| **PreP usage – Current and former PrEP users** | |
| Are you currently taking or have you ever taken drugs for pre-exposure prophylaxis (PrEP) against HIV? | - Yes, I am taking PrEP on a daily basis. - Yes, I take it intermittently when I think I need it - Yes, I used to take PrEP, but I permanently stopped - No |

| **General information – Current and former PrEP users** | |
| --- | --- |
| How old are you? | Drop-down (range 18 to >80) |
| What gender do you identify with? | - Male - Female - Trans* - Intersexual - Other |
| What is your approximate gross income per year?  *Gross income: income before deduction of taxes and social security contributions* | - Less than 30,000€ - 30,000 – 39,000€ - 40,000 – 49,000€ - 50,000 – 59,000€ - 60,000 – 69,000€ - 70,000€ or more - I don’t know |
| In which country were you born? | Drop-down |
| Where did you hear about this study? | Select all that apply   - Planetromeo - Grindr - Hornet - Checkpoint - through friends |
| With how many different male partners have you had anal sex within the last 6 months? | - 0 - 1 - 2-3 - 4-5 - 6-10 - more than 10 - I don’t know |
| How happy are you with your sex life at the moment? | - Very happy - Happy - I’m not sure - Unhappy - Very unhappy - Sex is not important for me at the moment |

| **Experiences with PrEP – Current PrEP users only** | |
| --- | --- |
| How are you taking PrEP? | - I am taking PrEP permanently - I am taking PrEP intermittently / occasionally (e.g. during pride season or on holidays) - I am taking PrEP on demand when I have risky sexual encounters - Other:______ |
| When did you first start taking PrEP? | - Less than 3 months ago - 3-6 months ago - 7-12 months ago - 13-24 months ago - More than 24 months ago |
| Within the last 12 months, for how many months did you take PrEP? | Drop-down: 0-12 months |
| How often do you take PrEP on average per month  If you take PrEP intermittently / occasionally please refer to the use of PrEP in a month when you are taking PrEP | - On all or almost all days (26 days or more) - On many or most days (12-25 days) - On a few days (1-11 days) |
| Where did you obtain PrEP from?  (please indicate the last source where you obtained PrEP) | - German pharmacy – prescription for about 50€ (Blister prescription) - German pharmacy prescription for more than 50€, but less than 100€ - German pharmacy – prescription for 500€ - 800€ - Friends - Dealer - Sex Party - Research Study - Internet / ordered online from another country - I regularly fly to another country where I get PrEP - I used PEP-medication as PrEP - Other: _________ |
| Since October 2017 PrEP in Germany is available at prices between 50€ and 70€ in pharmacies. Is the price affordable for you? | - Yes, I can afford PrEP - Yes, but it is hard for me to come up with the money to buy PrEP - No, I can’t afford PrEP at that price - I don’t know |

| **Clinical testing – Current PrEP users only** | |
| --- | --- |
| Have you gotten tested **before** starting PrEP medications (e.g. HIV test, STI tests, …)? | - Yes - No - I don’t know |
| Which of the following tests were administered to you **before starting PrEP**? | Select all that apply:   - Test for HIV - Hepatitis B - Hepatitis C - Syphilis - Gonorrhea - Chlamydia - Mycoplasma - Kidney function - Other tests   Don’t remember |
| Have you been tested for HIV, other sexually transmitted infections (STI), or have been checked for your kidney function **while taking PrEP**? | - Yes - No - Don’t know |
| **How often** do you get tested for HIV **while you are on PrEP**? | - At least once every 3 months - At least once every 6 months - At least once per year - Less than once every year - I don’t get tested for this - I don’t know |
| Why aren’t you getting tested for HIV?  Only if “not at all” was selected for previous question. | Select all that apply   - I can’t afford the test - The test was not offered to me - I don't have time to take the test - I don't want to take the test - I don't think I would benefit from this test - I didn’t know I was supposed to take this test - Other reason: |
| **How often** do you get tested for STIs **while you are on PrEP**, e.g. gonorrhoea, syphilis, chlamydia? | - At least once every 3 months - At least once every 6 months - At least once per year - Less than once every year - I don’t get tested for this - I don’t know |
| Why aren’t you getting tested for sexually transmitted infections (STI)?  Only if “not at all” was selected for previous question. | Select all that apply   - I can’t afford the tests - The tests were not offered to me - I don't have time to take the tests - I don't want to take the tests - I don't think I would benefit from these tests - I didn’t know I was supposed to take this test - Other reason: |
| For which of the following STIs have you tested positive? / Which STIs have you ever been diagnosed with? | Select all that apply:   - Syphilis - Gonorrhea - Chlamydia - Genital warts / HPV - Hepatitis A - Hepatitis B - Hepatitis C - Mycoplasma - Other: _____ - None - I don’t remember |
| Follow-up questions for every option in question 12 that was declared positive:   - When were you last diagnosed with a new syphilis infection? - When were you last tested positive for gonorrhea? - When were you last tested positive for chlamydia? - When were you first diagnosed with genital warts/ HPV? - When were you diagnosed with hepatitis A? - When were you diagnosed with hepatitis B? - When were you last diagnosed with hepatitis C? then: Did you ever have hepatitis C before (which either cleared spontaneously or was successfully treated)? - When were you last tested positive for mycoplasma? - When were you last tested positive for other: ____ | - Within the last 7 days - Within the last 4 weeks - Within the last 6 months - Within the last 12 months - Within the last 24 months - More than 24 months ago   Answers HCV-Followup Question   - No - Yes, once - Yes, more than once |
| **How often** do you get tested for your kidney function **while you are on PrEP**? | - At least once every 3 months - At least once every 6 months - At least once per year - Less than once every year - I am not getting tested for this - I don’t know |
| Why aren’t you getting a test to check your kidney function?  Only if “not at all” was selected for previous question | Check all that apply   - I can’t afford the test - The test was not offered to me - I don't have time to take the test - I don't want to take the test - I don't think I would benefit from this test - - I didn’t know I was supposed to take this test - other reason: |
| **Where** do you get tested while you are using PrEP (e.g. for HIV, STIs, or kidney function)? | Select all that apply:   - At the doctor who prescribes the medication - At a community based testing site / anonymous testing clinic - Other: ______ |
| **How much** do you pay when you get these tests? | - I don’t pay anything at the doctor / it’s covered by my health insurance - Less than 50€ for all tests - About 50-100€ for all tests - More than 100€ for all tests - I don’t know |

| **Drivers for PrEP use – Current PrEP users only** | |
| --- | --- |
| Why do you take PrEP? | Select all that apply:   - I don’t want to use condoms and still want to protect myself - My partner doesn’t want to use condoms and I still want to protect myself - Sex without a condom is expected by my peers and I still want to protect myself - I want to protect myself against HIV in case the condom breaks - Sometimes condoms aren’t available and I still want to be protected - It’s more convenient since I don’t have to talk about or negotiate condom use - I cannot get an erection when I use a condom but I still want to protect myself - My partner is HIV positive and I want to protect myself - I use condoms and I want additional protection - Other: _________ |

| **Drivers for PrEP use – Current PrEP users only** | |
| --- | --- |
| How often do you use condoms for anal sex in periods when you are taking PrEP? | - Always - Often - About half of the times I have anal sex - Sometimes - Never - I don’t know |
| Since I am taking PrEP, I am using condoms for anal sex… | - More often than before - As often as before - Less often than before - I have stopped using condoms altogether - I don’t know |
| Do you indicate on your online profile(s) that you are taking PrEP? | - Yes - No, but I mention it when I chat with other guys - No |

| **Questions on former PrEP use – Former PrEP users only** | |
| --- | --- |
| Why did you stop using PrEP? | Select all that apply:   - I feel safe enough with other prevention strategies (condoms, etc) - I don’t need it because I am currently not having sex - I feel I don’t need it because I have fewer sexual partners than before - I feel I don’t need it anymore because I trust my partner / partners - I don’t want to take a daily pill - I experienced side effects - I am worried about long-term side effects with PrEP - I had too many STIs while I was having condomless sex on PrEP - I would like to use PrEP but the price is too high for me (50-70€ per month) - I would like to use PrEP but I cannot get it any more through my original source (e.g. clinical trial ended, dealer changed, friends do not share PrEP any more) - I would like to use PrEP but I’m afraid of stigma against PrEP users by others / my partner - I tested positive for HIV - I think that using PrEP is immoral and/or irresponsible - I don’t want to unnecessarily expose my body to chemicals - Other ____ |
| How did you use PrEP? | - I was taking PrEP permanently - I was taking PrEP intermittently / occasionally (e.g. during pride season or on holidays) - I was taking PrEP on demand only when I was having risky sexual encounters - Other:____ |
| For how long have you been using PrEP? | - Less than 3 months - 3-6 months - 7-12 months - 13-24 months - Longer than 24 months |
| How often did you use PrEP on average per month?  *If you used PrEP intermittently / occasionally please refer to the use of PrEP in a month when you were taking PrEP* | - On all or almost all days (26 days or more) - On many or most days (12-25 days) - On a few days (1-11 days) |
| How often do you use condoms to prevent HIV infection during anal sex since stopping PrEP? | - Always - Often - In about half of the times I had sex - Sometimes - Never - I don’t know |

## Appendix S2: Recruitment of participants


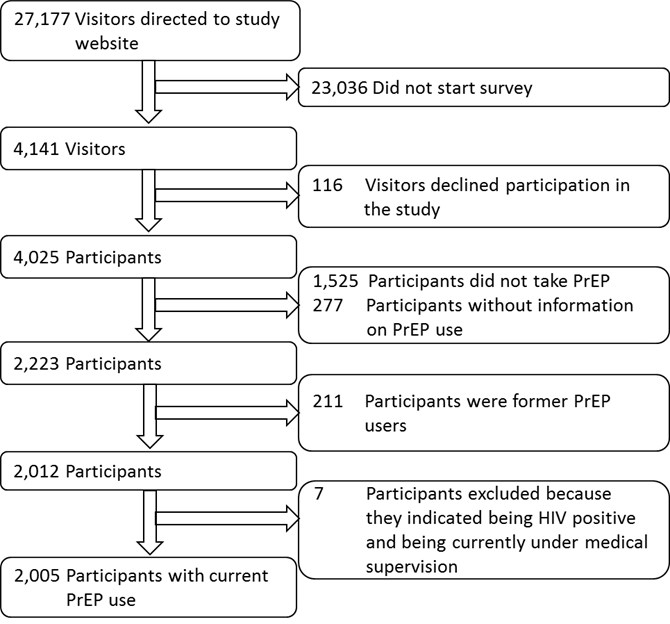


## Appendix S3: Supplemental data on participants with formal and informal PrEP use

|  | Prescription PrEP n (%) | Informal PrEP n (%) |
| --- | --- | --- |
|  |  |  |
| Total | 1,436 (100) | 348 (100) |
| Average number of pills taken per month |  |  |
| ≥ 26 days | 1,054 (73.4) | 149 (42.8) |
| < 26 days | 282 (19.6) | 159 (45.7) |
| Missing | 100 (7.0) | 40 (11.5) |
| Development of condom use since starting PrEP |  |  |
| More often than before | 15 (1.0) | 4 (1.2) |
| As often as before | 273 (19.0) | 64 (18.4) |
| Less often than before | 736 (51.3) | 158 (45.4) |
| Stopped using condoms completely | 301 (21.0) | 79 (22.7) |
| I don’t know / Missing | 111 (7.7) | 43 (12.4) |

## Appendix S4: Investigating factors associated with informal PrEP use excluding participants receiving PrEP through clinical trials

|  | Prescription PrEP n (%) | Informal PrEP n (%) | Univariable Analysis^†^ | | Multivariable Analysis^‡^ | |
| --- | --- | --- | --- | --- | --- | --- |
|  |  |  | **OR (95% CI)** | ***p*-value** | **OR (95% CI)** | ***p*-value** |
| Total | 1,360 | 348 |  |  |  |  |
| Time since first PrEP use |  |  |  |  |  |  |
| < 6 months | 682 (50.2) | 91 (26.2) | 1 |  | 1 |  |
| ≥ 7 months | 593 (43.6) | 218 (62.6) | 2.7 (2.0 – 3.7) | <0.001 | 2.8 (2.0 – 4.0) | <0.001 |
| Missing | 85 (6.3) | 39 (11.2) | - |  |  |  |
| Type of PrEP use |  |  |  |  |  |  |
| Daily | 969 (71.3) | 139 (39.9) | 1 |  | 1 |  |
| On demand / intermittent | 309 (22.7) | 168 (48.3) | 4.1 (3.0 – 5.5) | <0.001 | 3.3 (2.3 – 4.7) | <0.001 |
| Missing | 82 (6.0) | 41 (11.8) | - |  |  |  |
| Tests before starting PrEP (e.g. HIV, STI, kidney function) |  |  |  |  |  |  |
| Yes | 1,316 (96.8) | 289 (83.1) | 1 |  | 1 |  |
| No | 27 (2.0) | 43 (12.4) | 7.0 (3.7 – 13.2) | <0.001 | 3.0 (1.4 – 6.5) | 0.007 |
| Missing | 17 (1.3) | 16 (4.6) | - |  |  |  |
| Tests while taking PrEP (e.g. HIV, STI, kidney function) |  |  |  |  |  |  |
| Yes | 1,200 (88.2) | 223 (64.1) | 1 |  | 1 |  |
| No | 96 (7.1) | 96 (27.6) | 5.8 (4.0 – 8.4) | <0.001 | 3.2 (2.0 – 5.0) | <0.001 |
| Missing | 64 (4.7) | 29 (8.3) | - |  |  |  |
| Country of origin (%) |  |  |  |  |  |  |
| Germany | 868 (63.8) | 164 (47.1) | 1 |  | 1 |  |
| Outside Germany | 252 (18.5) | 99 (28.5) | 2.1 (1.5 – 2.8) | <0.001 | 2.3 (1.6 – 3.3) | <0.001 |
| Missing | 240 (17.7) | 85 (24.4) | - |  |  |  |
| Number of anal sex partners within the last 6 months, n (%) |  |  |  |  |  |  |
| 0 – 3 | 180 (13.2) | 49 (14.1) | 1.2 (0.8 – 1.9) | 0.431 | 1.0 (0.6 – 1.8) | 0.870 |
| 4 – 10 | 406 (29.9) | 90 (25.9) | 1 |  | 1 |  |
| > 10 | 665 (48.9) | 164 (47.1) | 1.1 (0.8 – 1.6) | 0.417 | 1.2 (0.8 – 1.7) | 0.415 |
| Missing | 109 (8.0) | 45 (12.9) | - |  |  |  |
| Condom use while taking PrEP |  |  |  |  |  |  |
| Always / often | 280 (20.6) | 55 (15.8) | 1 |  | 1 |  |
| In about half of the times /  sometimes / never | 982 (72.2) | 248 (71.3) | 1.3 (0.9 – 2.0) | 0.120 | 1.4 (0.9 – 2.3) | 0.119 |
| Missing | 98 (7.2) | 45 (12.9) | - |  |  |  |
| Is PrEP for a price of 50-70€ per month affordable? |  |  |  |  |  |  |
| Yes | 907 (66.7) | 183 (52.6) | 1 |  | 1 |  |
| Yes, but it is difficult to manage | 400 (29.4) | 91 (26.2) | 1.4 (1.0 – 1.9) | 0.064 | 1.6 (1.0 – 2.4) | 0.045 |
| No | 39 (2.9) | 57 (16.4) | 8.8 (5.1 – 15.1) | <0.001 | 8.8 (4.6 – 17.1) | <0.001 |
| Missing | 14 (1.0) | 17 (4.9) | - |  |  |  |
| Communication of PrEP on online profile |  |  |  |  |  |  |
| Yes | 610 (44.9) | 131 (37.6) | 1 |  | 1 |  |
| No, but mentions it while chatting | 436 (32.1) | 122 (35.1) | 1.3 (0.9 – 1.8) | 0.123 | 1.0 (0.7 – 1.4) | 0.870 |
| No | 212 (15.6) | 53 (15.2) | 1.1 (0.7 – 1.7) | 0.609 | 0.9 (0.5 – 1.5) | 0.600 |
| Missing | 102 (7.5) | 42 (12.1) | - |  |  |  |
| Age |  |  |  |  |  |  |
| 18 – 29 years | 242 (17.8) | 77 (22.1) | 1.4 (0.9 – 2.0) | 0.094 | 0.9 (0.6 – 1.5) | 0.784 |
| 30 – 39 years | 484 (35.6) | 92 (26.4) | 1 |  | 1 |  |
| 40 – 49 years | 357 (26.3) | 98 (28.2) | 1.2 (0.8 – 1.7) | 0.334 | 1.3 (0.8 – 1.9) | 0.248 |
| 50 – 80 years | 171 (12.6) | 35 (10.1) | 0.9 (0.5 – 1.4) | 0.552 | 0.8 (0.4 – 1.4) | 0.378 |
| Missing | 106 (7.8) | 46 (13.2) | - |  |  |  |
| Annual gross income, n (%) |  |  |  |  |  |  |
| <30,000 € | 297 (21.8) | 79 (22.7) | 1 |  | 1 |  |
| 30,000 – 39,000 € | 207 (15.2) | 55 (15.8) | 0.8 (0.5 – 1.2) | 0.215 | 1.6 (0.9 – 2.7) | 0.097 |
| 40,000 – 49,000 € | 182 (13.4) | 35 (10.1) | 0.7 (0.4 – 1.1) | 0.128 | 1.3 (0.7 – 2.3) | 0.461 |
| 50,000 – 59,000 € | 138 (10.2) | 27 (7.8) | 0.7 (0.4 – 1.1) | 0.122 | 1.7 (0.8 – 3.3) | 0.137 |
| 60,000 – 69,000 € | 104 (7.7) | 31 (8.9) | 1.2 (0.7 – 1.9) | 0.538 | 2.4 (1.2 – 4.6) | 0.011 |
| ≥70,000 € | 256 (18.8) | 55 (15.8) | 0.8 (0.5 – 1.2) | 0.298 | 1.7 (0.9 – 3.0) | 0.088 |
| Missing | 176 (12.9) | 66 (19.0) | - |  |  |  |

^†^Univariable logistic regression model including 1034 participants with formal and 230 participants with informal PrEP use, p-values derived from Wald test

^‡^Multivariable logistic regression model including 1034 participants with formal and 230 participants with informal PrEP use adjusting for age, annual gross income, country of origin, type of PrEP use, time since first PrEP use, affordability of generic PrEP, tests before starting PrEP, tests during PREP use, number of anal sex partners within the last 6 months, condom use, communication of PrEP use online; p-values: Wald test

CI: Confidence Interval, OR: Odds Ratio, PrEP: Pre-exposure prophylaxis, HIV: human immunodeficiency virus, STI: sexually transmitted infection

**Stratum-specific Odds Ratios of multivariable logistic regression analysis investigating factors associated with informal PrEP use**^†^

|  | Daily PrEP use | | On Demand / intermittent PrEP use | | Likelihood-Ratio test for interaction |
| --- | --- | --- | --- | --- | --- |
|  | **OR (95% CI)** | ***p*-value** | **OR (95% CI)** | ***p*-value** | ***p*-value** |
| Tests before starting PrEP (e.g. HIV, STI, kidney function) |  |  |  |  |  |
| Yes | 1 |  | 1 |  | 0.009 |
| No | 0.5 (0.1 – 3.0) | 0.480 | 34.0 (4.8 – 240.5) | <0.001 |  |
| Tests while taking PrEP (e.g. HIV, STI, kidney function) |  |  |  |  |  |
| Yes | 1 |  | 1 |  | 0.990 |
| No | 3.2 (1.4 – 7.1) | 0.005 | 3.3 (1.3 – 8.0) | 0.009 |  |
| Is PrEP for a price of 50-70€ per month affordable? |  |  |  |  |  |
| Yes | 1 |  |  |  | 0.030 |
| Yes, but it is difficult to manage | 2.0 (1.2 – 3.5) | 0.009 | 2.4 (1.3 – 4.3) | 0.005 |  |
| No | 16.7 (7.0 – 40.0) | <0.001 | 1.1 (0.4 – 3.5) | 0.805 |  |

^†^Multivariable logistic regression model including 1034 participants with formal and 230 participants with informal PrEP use adjusting for age, annual gross income, country of origin, type of PrEP use, time since first PrEP use, affordability of generic PrEP, tests before starting PrEP, tests during PREP use, number of anal sex partners within the last 6 months, condom use, communication of PrEP use online; p-values: Wald test

CI: Confidence Interval, OR: Odds Ratio, PrEP: Pre-exposure prophylaxis, HIV: human immunodeficiency virus, STI: sexually transmitted infection

## Appendix S5: Investigating factors associated with informal PrEP use excluding participants ordering informal PrEP through online sources

|  | Prescription PrEP n (%) | Informal PrEP n (%) | Univariable Analysis^†^ | | Multivariable Analysis^‡^ | |
| --- | --- | --- | --- | --- | --- | --- |
|  |  |  | **OR (95% CI)** | **p-value** | **OR (95% CI)** | **p-value** |
| Total | 1,436 (100) | 171 |  |  |  |  |
| Time since first PrEP use |  |  |  |  |  |  |
| < 6 months | 686 (47.8) | 45 (26.3) | 1 |  | 1 |  |
| ≥ 7 months | 656 (45.7) | 103 (60.2) | 2.2 (1.4 – 3.4) | <0.001 | 2.6 (1.6 – 4.3) | <0.001 |
| Missing | 94 (6.6) | 23 (13.5) | - |  |  |  |
| Type of PrEP use |  |  |  |  |  |  |
| Daily | 1,034 (72.0) | 62 (36.3) | 1 |  | 1 |  |
| On demand / intermittent | 311 (21.7) | 86 (50.3) | 5.1 (3.3 – 7.7) | <0.001 | 3.3 (2.0 – 5.5) | <0.001 |
| Missing | 91 (6.3) | 23 (13.5) | - |  |  |  |
| Tests before starting PrEP (e.g. HIV, STI, kidney function) |  |  |  |  |  |  |
| Yes | 1,391 (96.9) | 136 (79.5) | 1 |  | 1 |  |
| No | 27 (1.9) | 21 (12.3) | 6.0 (2.6 – 13.9) | <0.001 | 2.1 (0.8 – 6.0) | 0.149 |
| Missing | 18 (1.3) | 14 (8.2) | - |  |  |  |
| Tests while taking PrEP (e.g. HIV, STI, kidney function) |  |  |  |  |  |  |
| Yes | 1,274 (88.7) | 96 (56.1) | 1 |  | 1 |  |
| No | 96 (6.7) | 57 (33.3) | 8.7 (5.5 – 13.9) | <0.001 | 4.3 (2.4 – 7.7) | <0.001 |
| Missing | 66 (4.6) | 18 (10.5) | - |  |  |  |
| Country of origin (%) |  |  |  |  |  |  |
| Germany | 913 (63.6) | 67 (39.2) | 1 |  | 1 |  |
| Outside Germany | 269 (18.7) | 56 (32.7) | 3.0 (2.0 – 4.5) | <0.001 | 3.0 (1.8 – 4.9) | <0.001 |
| Missing | 254 (17.7) | 48 (28.1) | - |  |  |  |
| Number of anal sex partners within the last 6 months, n (%) |  |  |  |  |  |  |
| 0 – 3 | 190 (13.2) | 32 (18.7) | 1.3 (0.7 – 2.4) | 0.364 | 1.3 (0.6 – 2.5) | 0.521 |
| 4 – 10 | 417 (29.0) | 46 (26.9) | 1 |  | 1 |  |
| > 10 | 711 (49.5) | 66 (38.6) | 0.8 (0.5 – 1.3) | 0.412 | 1.0 (0.6 – 1.7) | 0.936 |
| Missing | 118 (8.2) | 27 (15.8) | - |  |  |  |
| Condom use while taking PrEP |  |  |  |  |  |  |
| Always / often | 283 (19.7) | 37 (21.6) | 1 |  | 1 |  |
| In about half of the times /  sometimes / never | 1,046 (72.8) | 107 (62.6) | 0.8 (0.5 – 1.3) | 0.390 | 1.0 (0.5 – 1.7) | 0.911 |
| Missing | 107 (7.5) | 27 (15.8) | - |  |  |  |
| Is PrEP for a price of 50-70€ per month affordable? |  |  |  |  |  |  |
| Yes | 950 (66.2) | 77 (45.0) | 1 |  | 1 |  |
| Yes, but it is difficult to manage | 417 (29.0) | 52 (30.4) | 2.3 (1.5 – 3.5) | <0.001 | 2.3 (1.3 – 4.3) | 0.006 |
| No | 49 (3.4) | 28 (16.4) | 8.9 (4.6 – 17.3) | <0.001 | 6.2 (2.7 – 14.7) | <0.001 |
| Missing | 20 (1.4) | 14 (8.2) | - |  |  |  |
| Communication of PrEP on online profile |  |  |  |  |  |  |
| Yes | 655 (45.6) | 50 (29.2) | 1 |  | 1 |  |
| No, but mentions it while chatting | 453 (31.6) | 67 (39.2) | 1.9 (1.2 – 2.9) | 0.007 | 1.2 (0.7 – 2.1) | 0.458 |
| No | 217 (15.1) | 29 (17.0) | 1.5 (0.8 – 2.7) | 0.173 | 1.0 (0.5 – 2.1) | 0.993 |
| Missing | 111 (7.7) | 25 (14.6) | - |  |  |  |
| Age |  |  |  |  |  |  |
| 18 – 29 years | 253 (17.6) | 53 (31.0) | 2.2 (1.3 – 3.7) | 0.002 | 1.4 (0.8 – 2.5) | 0.291 |
| 30 – 39 years | 514 (35.8) | 40 (23.4) | 1 |  | 1 |  |
| 40 – 49 years | 377 (26.3) | 35 (20.5) | 1.0 (0.6 – 1.7) | 0.965 | 1.1 (0.6 – 2.0) | 0.791 |
| 50 – 80 years | 177 (12.3) | 15 (8.8) | 0.8 (0.4 – 1.8) | 0.605 | 0.8 (0.3 – 1.8) | 0.548 |
| Missing | 115 (8.0) | 28 (16.4) | - |  |  |  |
| Annual gross income, n (%) |  |  |  |  |  |  |
| <30,000 € | 320 (22.3) | 46 (26.9) | 1 |  | 1 |  |
| 30,000 – 39,000 € | 217 (15.1) | 30 (17.5) | 0.7 (0.4 – 1.3) | 0.270 | 1.7 (0.8 – 3.4) | 0.135 |
| 40,000 – 49,000 € | 191 (13.3) | 14 (8.2) | 0.4 (0.2 – 0.9) | 0.026 | 1.0 (0.4 – 2.4) | 0.994 |
| 50,000 – 59,000 € | 144 (10.0) | 10 (5.8) | 0.3 (0.1 – 0.8) | 0.015 | 1.1 (0.4 – 3.1) | 0.904 |
| 60,000 – 69,000 € | 109 (7.6) | 6 (3.5) | 0.4 (0.1 – 1.0) | 0.051 | 1.0 (0.3 – 3.2) | 0.965 |
| ≥70,000 € | 265 (18.5) | 25 (14.6) | 0.7 (0.4 – 1.2) | 0.171 | 2.3 (1.0 – 5.2) | 0.039 |
| Missing | 190 (13.2) | 40 (23.4) | - |  |  |  |

^†^Univariable logistic regression model including 1089 participants with formal and 103 participants with informal PrEP use, p-values derived from Wald test

^‡^Multivariable logistic regression model including 1089 participants with formal and 103 participants with informal PrEP use adjusting for age, annual gross income, country of origin, type of PrEP use, time since first PrEP use, affordability of generic PrEP, tests before starting PrEP, tests during PREP use, number of anal sex partners within the last 6 months, condom use, communication of PrEP use online; p-values: Wald test

CI: Confidence Interval, OR: Odds Ratio, PrEP: Pre-exposure prophylaxis, HIV: human immunodeficiency virus, STI: sexually transmitted infection

**Stratum-specific Odds Ratios of multivariable logistic regression analysis investigating factors associated with informal PrEP use**^†^

|  | Daily PrEP use | | On Demand / intermittent PrEP use | | Likelihood-Ratio test for interaction |
| --- | --- | --- | --- | --- | --- |
|  | **OR (95% CI)** | **p-value** | **OR (95% CI)** | **p-value** | **p-value** |
| Tests before starting PrEP (e.g. HIV, STI, kidney function) |  |  |  |  |  |
| Yes | 1 |  | 1 |  | 0.051 |
| No | 0.4 (0.0 – 4.2) | 0.478 | 31.5 (2.4 – 413.2) | 0.009 |  |
| Tests while taking PrEP (e.g. HIV, STI, kidney function) |  |  |  |  |  |
| Yes | 1 |  | 1 |  | 0.618 |
| No | 5.3 (2.0 – 14.1) | 0.001 | 2.6 (0.9 – 7.5) | 0.066 |  |
| Is PrEP for a price of 50-70€ per month affordable? |  |  |  |  |  |
| Yes | 1 |  | 1 |  | 0.046 |
| Yes, but it is difficult to manage | 3.5 (1.6 – 7.8) | 0.002 | 2.5 (1.2 – 5.3) | 0.016 |  |
| No | 15.1 (5.0 – 46.0) | <0.001 | 1.0 (0.3 – 4.0) | 0.983 |  |

^†^Multivariable logistic regression model including 1089 participants with formal and 103 participants with informal PrEP use adjusting for age, annual gross income, country of origin, type of PrEP use, time since first PrEP use, affordability of generic PrEP, tests before starting PrEP, tests during PREP use, number of anal sex partners within the last 6 months, condom use, communication of PrEP use online; p-values: Wald test

CI: Confidence Interval, OR: Odds Ratio, PrEP: Pre-exposure prophylaxis, HIV: human immunodeficiency virus, STI: sexually transmitted infection
